# Supplementary material for: Purification and Characterization of Bot33: A Non-Toxic Peptide from the Venom of Buthus occitanus tunetanus Scorpion
Source: Molecules. 2022 Oct 26;27(21):7278. doi: 10.3390/molecules27217278 (PMC9657394; doi:10.3390/molecules27217278)
Supplement: Supplementary file 1 [file molecules-27-07278-s001.zip › molecules-1862695-supplementary.pdf]

Supplementary data

Figure S1: A real photo of a scorpion: *Buthus occitanus tunetanus*

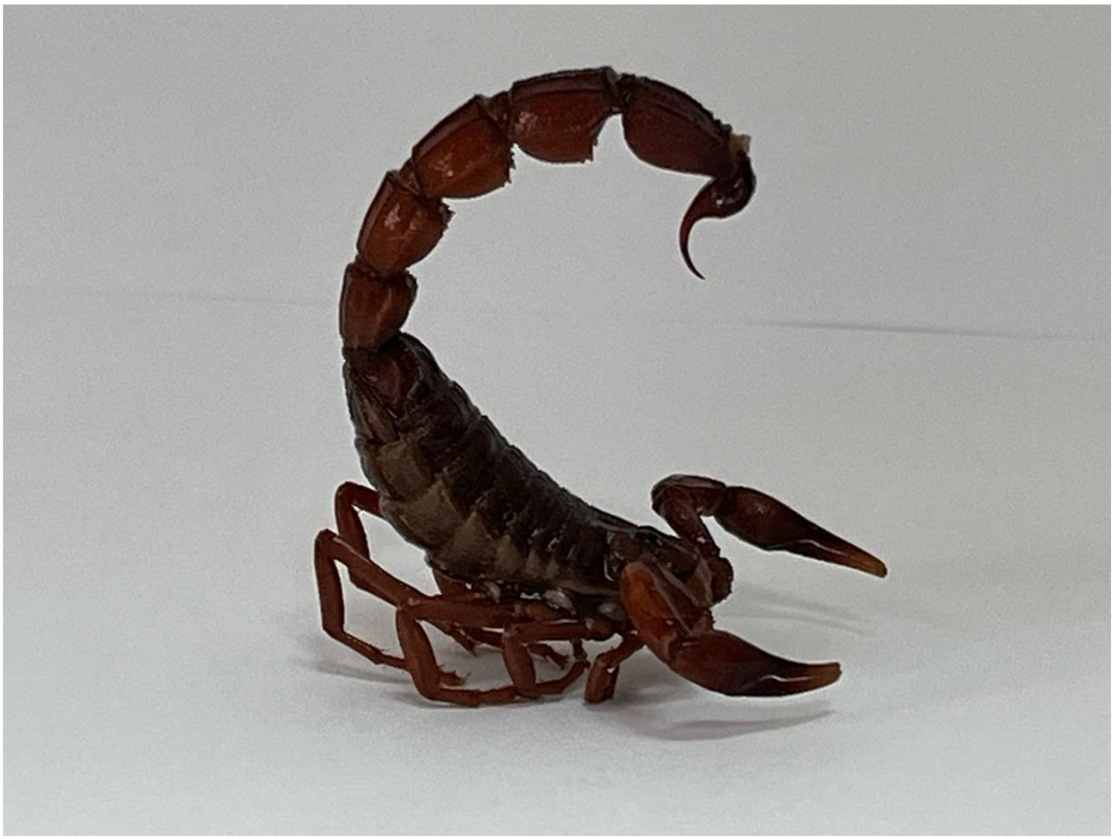

Table S1 : *In vivo* toxicity tests: increasing amounts of native and synthetic Bot33 were performed by i.c.v. injection routes into black mice model. Toxicity symptoms were monitored during 24h.

Native and synthetic Bot33 concentrations (ng/mouse)	Time (h)	Mice (death/total)
10	24	0/6
15	24	0/6
20	24	0/6
25	24	0/6
30	24	0/6
37	24	0/6
